# Thermographic Inspection of Internal Defects in Steel Structures: Analysis of Signal Processing Techniques in Pulsed Thermography

**DOI:** 10.3390/s20216015

**Published:** 2020-10-23

**Authors:** Yoonjae Chung, Ranjit Shrestha, Seungju Lee, Wontae Kim

**Affiliations:** 1 Department of Mechanical Engineering, Kongju National University, 1223-24 Cheonan-daero, Seobuk-gu, Cheonan-si 31080, Korea; dbswosla79@kongju.ac.kr; 2 Department of Mechanical Engineering, School of Engineering, Kathmandu University, Dhulikhel, P.O. Box, Kathmandu 6250, Nepal; ranjit.shrestha@ku.edu.np; 3 Department of Convergence Mechanical Engineering, Kongju National University, 1223-24 Cheonan-daero, Seobuk-gu, Cheonan-si 31080, Korea; cow123456798@smail.kongju.ac.kr; 4 Division of Mechanical & Automotive Engineering, Kongju National University, 1223-24 Cheonan-daero, Seobuk-gu, Cheonan-si 31080, Korea

**Keywords:** internal defects, nondestructive testing, pulsed thermography, thermal signal reconstruction, pulsed phase thermography, principle component analysis, signal to noise ratio

## Abstract

This study performed an experimental investigation on pulsed thermography to detect internal defects, the major degradation phenomena in several structures of the secondary systems in nuclear power plants as well as industrial pipelines. The material losses due to wall thinning were simulated by drilling flat-bottomed holes (FBH) on the steel plate. FBH of different sizes in varying depths were considered to evaluate the detection capability of the proposed technique. A short and high energy light pulse was deposited on a sample surface, and an infrared camera was used to analyze the effect of the applied heat flux. The three most established signal processing techniques of thermography, namely thermal signal reconstruction (TSR), pulsed phase thermography (PPT), and principal component thermography (PCT), have been applied to raw thermal images. Then, the performance of each technique was evaluated concerning enhanced defect detectability and signal to noise ratio (SNR). The results revealed that TSR enhanced the defect detectability, detecting the maximum number of defects, PPT provided the highest SNR, especially for the deeper defects, and PCT provided the highest SNR for the shallower defects.

## 1. Introduction

Steel is one of the world’s most versatile materials with excellent qualities of strength, corrosion resistance, weldability, formability, workability, and attractiveness. Also, having a long-life cycle and being 100% recyclable, it is the ideal material for a wide range of applications, such as architecture and construction, automotive and transportation, aircraft, chemical, food and beverage, energy, medical, and a variety of other applications. Steel is used in the construction of reactor vessels and vessel liners, reactor building structure, pressure channels, fuel cladding, heat exchanger and condenser tubes, and fuel pool liners in the nuclear industry as well as industrial pipelines [1,2,3]. A major challenge seen in these systems is the wall thinning due to flow accelerated corrosion and liquid droplet impingement. Such degradation mechanism affects the structural health and can lead to disastrous failures of the structure; as well as a massive economic loss [4,5,6]. This calls for a reliable testing and evaluation method to ensure that the functionality of the system is not compromised due to thickness reduction. Recently, numerous nondestructive testing (NDT) techniques, such as x-ray [7,8,9], microwave [10,11,12], ultrasonic [13,14,15,16], magnetic flux leakage [17,18,19], acoustic emission [20,21,22], and eddy current [23,24,25], have been used for the measurement of pipe wall thinning. Each of these techniques have their own advantages and limitations. The lists of NDT techniques are continuously increasing as researchers around the globe are striving for the development of techniques and methods to guarantee market superiority in terms of quality and productivity. In this context, infrared thermography (IRT) plays a dynamic role to improve the quality and productivity of a scheme from creation to completion.

**Figure 1:**
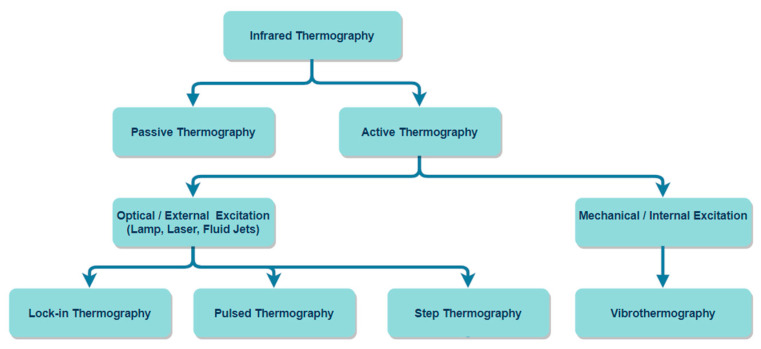
Classification of common infrared thermography techniques used in NDT.

IRT is an optical measurement technique, evolving rapidly with the development of high spatial resolution and sensitivity detectors and improved computation power [26,27,28]. IRT has been gaining increased recognition in recent years because of its peculiar advantages over its counterparts. These advantages include high-speed contactless functioning, higher level of safety, and portability with a potential to encompass a large inspection area [29,30,31]. IRT utilizes an infrared camera to extract and analyse a thermal pattern based on the principle that all objects above absolute zero (−273 °C) emit infrared energy. The infrared radiation emitted by an object is sensed by the infrared camera and transformed into an electronic signal, which is then processed to produce a thermal image [32]. As shown in Figure 1, there exist several types of thermographic approaches which can be classified based on the procedure for testing the structural components, the origin of the source of excitation energy, and the temperature differences of the constituents. Based on the experimental procedure, thermography can be classified into active and passive thermography. Passive thermography does not require any external source to excite surface thermal gradients as it is naturally at a higher or lower temperature compared to the background. In contrast, active thermography utilizes an external heating source to produce thermal contrast in the region of interest. Based on the exciting method, active thermography can be further classified as pulsed thermography (PT), lock-in thermography (LIT), vibrothermography (VT), and step heating thermography (SHT) [33,34,35]. PT and LIT are the two primary conventional practices. In both techniques, thermal energy is delivered into the object in which the heat propagates by diffusion through the material. Then, the thermal response recorded by the infrared camera is observed to reveal the presence of the defect. The experiment can be performed in two distinct ways. In transmission mode, an infrared camera and the excitation source are kept opposite the sample under investigation. In contrast, in reflection mode, an infrared camera and the excitation source are positioned identically to the sample being investigated [36,37]. Furthermore, various signal processing and filtering techniques can be used to access defects information, including the small internal discontinuities and material characteristics.

In the past few decades, research has demonstrated IRT to be useful in detection, identification, and classification of defects in composites, metals, polymers as well as ceramic materials. G. Busse et al. [38,39] proposed a four-point method for LIT to extract amplitude and phase angle images and also demonstrated that the phase angle image at each pixel provides the depth information of flat bottom holes (FBH) drilled from the rear side in a polymer as well as in inhomogeneous and anisotropic materials such as CFRP. D. Wu and G. Busse [40] later revealed that a phase angle image can eliminate the influences of uneven heating, surrounding reflections, and local disruptions like infrared emission coefficient and surface optical absorption. B.B. Lahri et al. [41] investigated the quantification of artificially produced FBH defects in composite, rubber, and aluminium structures using LIT and concluded that defects with a radius to depth ratio greater than 1.0 are detectable, which was in agreement with the empirical rule of thumb developed by V. Vavilov and R. Taylor [42]. C. Wallbrink et al. [43] observed the influence of defect size on the assessment of defect depth on a steel structure with FBH using LIT and concluded that the defect size had a significant effect on the phase angle which subsequently has consequences on the defect depth. C. Manyong et al. [44] and L. Junyan et al. [45] used LIT to evaluate the FBH defects in steel structure and showed that the phase angle differences between the defective regions and sound regions provide the defect location and size. D. Sharath et al. [46] studied on the blind frequency and phase contrast methods to evaluate the artificial square, rectangular and circular defects in austenitic stainless steel using LIT. It was concluded that blind frequency is dependent on the defect size and shape and could not be applied for depth quantification unless a normalization method is used. It was also concluded that phase contrast is a function of defect size and shape, apart from depth. S. Ranjit [47] investigated the effect of modulation frequency on the defect detectability on stainless steel with artificial FBH defects using LIT and concluded that the correct excitation frequency range must be selected for the inspected material to detect the defects. Although there has been increasing attention with LIT based structural health monitoring, longer observation time could be the main disadvantage of LIT as the experiment needs to be performed at multiple frequencies. To address this shortcoming, the PT played the important role in NDT. X. Maldague et al. [48] used the PT to inspect different kinds of defects in aluminum structures and showed that the heat pulse of energy ranging from 5 kJ to 20 kJ is enough to excite the object under inspection and emphasize on the frame rate with a high frequency to resolve the thermal process. C. Deemer [49] used temperature contrast method based PT to examine the carbon/carbon and continuous fiber ceramic composite components with drilled FBH at the back surface. C. Ibarra-Castanedo et al. [50] investigated the fact that PT raw data is commonly affected by problems, such as uneven heating, emissivity variations, reflections from the environment, and surface geometry sharp variations. Nevertheless, a wide variety of PT data processing techniques were developed to carry out qualitative and quantitative inspections. O. Wysocka-Fotek et al. [51] used standard thermal contrast method based on PT to estimate the size and depth of FBH defects in austenitic steel. X. Maldague et al. [52,53] combined the facets of PT and LIT techniques and proposed pulsed phase thermography (PPT). It was demonstrated that PPT can detect the deeper defects with other features such as less influence of optical characteristics and surface infrared, and the possibility to inspect test sample with higher thermal conductivity. S.M. Shepard et al. [54] proposed the thermal signal reconstruction (TSR) technique and analyzed the experimental PT data to detect the FBH defects in steel structure. It was also concluded that the time derivative of the reconstructed data detects the defect with high sensitivity at earlier times with better SNR. N. Rajic [55] applied a singular value decomposition method based principal component thermography (PPT) to the NDT of the composite structure and demonstrated the evidence of reduction in noise to a considerable extent with the high level of defect contrast. A. Vageswar et al. [56] investigated the peak contrast slope method using PT in transmission mode to estimate the depth of FBH defects in steel structures. Z. Zeng et al. [57] used PT to investigate the steel sample with FBH defects filled with different materials to replicate different non-air interfaces. The comparison between peak slope time method, absolute peak slope time method, logarithmic peak second-derivative method, and peak contrast time method showed that the later one is extremely influenced by defect size and interface while the other three techniques provided accurate defect depth. P. Simon and A. Darryl [58] used PT and LIT techniques to detect the FBH defects in CFRP and compared their results with respect to SNR. The comparison revealed the better performance of PT with a higher SNR for shallower defects. However, in the case of deeper defects, both the PT and LIT provided approximately the same SNR. D. Duan [59] investigated the detection capability and reliability of PT and LIT techniques to detect FBH defects in aluminium foams introducing probability of detection analysis. C. Krishnendu et al. [60] researched the testing of carbon fibre composite sample using PT, LIT, and modulated thermography, and the results of each technique were compared in terms of SNR. It was concluded that PT provided higher SNR for the shallower defects whereas for the deeper defects all the three techniques provided the approximately same SNR. This fact showed that the selection of the most adequate IRT approach depends on the particular application and the available experimental and expertise resources.

In this work, an experimental thermographic inspection process of detecting wall thinning defects in steel structure is presented. A test sample with FBH was considered as a best-case scenario to represent real wall thinning defects as the difference between real wall thinning defects and FBH defects are relatively small. The primary purpose of the study was to investigate the capabilities of the PT to detect the defects in the referenced sample. The second purpose was to employ the standard signal processing techniques, namely, TSR, PPT, and PCT, and then evaluate their performance in terms of defect detectability and SNR.

The remaining part of the research article is arranged as per the followings: Section 2 introduces a quick review of PT. Section 3 provides the principle and mathematical representation of signal processing techniques, namely, TSR, PPT and PCT. Section 4 presents the details of the test sample and experimentation. Section 5 discusses the results and analysis of each signal processing technique. Finally, Section 6 outlines the conclusions and possible future works.

## 2. Pulsed Thermography

**Figure 2:**
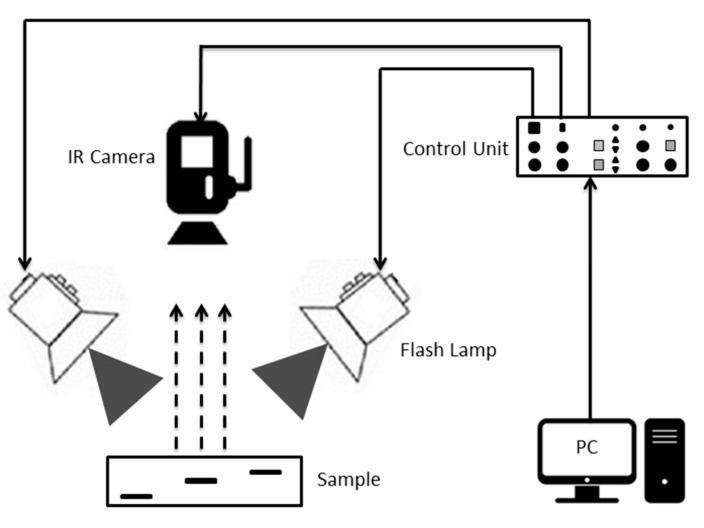
Experimental configuration of a pulsed thermography inspection system.

Pulsed thermography (PT) is among the most prevalent thermographic techniques in NDT. Figure 2 depicts the principle of PT where a high-power pulse heating is employed to the sample under inspection and the response of the sample is recorded with an infrared camera. The continuance of the pulse depends on the thermal conductivity of the sample under inspection and runs between 2 milliseconds to 10 milliseconds, making PT well known for its quickness in testing. As soon as the sample got excited, the surface absorbs the light energy and temperature increases instantly. Due to the propagation of a thermal wave inside the sample, the surface temperature starts to decay. Imperfections could be seen if there is variance in the thermal decay rate across the sample surface [61,62,63].

The one-dimensional solution of the Fourier equation for the propagation of a Dirac heat pulse in a semi-infinite isotropic solid by conduction can be expressed by Equation (1) [64].

(1) $$T\left(z,t\right)=T_{0}+\frac{Q}{e\sqrt{\pi t}}\exp\left(-\frac{z^{2}}{4\alpha t}\right)$$

where T (K) is the temperature rise at the time (t) after the flash heating, $T_{0}$ (K) the initial temperature, Q (W/m^2^) the energy deposited on the surface, $e=\sqrt{{k\rho c}_{p}}$ (J/s^1/2^m^2^K) is the material’s thermal effusivity to exchange heat with its surrounding, $\alpha =\frac{k}{\rho .C_{p}}$ (m^2^/s) is the material’s thermal diffusivity with k (W/m.K) being the thermal conductivity, ρ (kg/m^3^) the density, and C_p_ (J/kg.K) the specific heat capacity.

At the surface (z = 0 mm), Equation (1) can be rewritten as Equation (2) [65].

(2) $$T\left(t\right)=\frac{Q}{e\sqrt{\pi t}}$$

## 3. Signal Processing Techniques

PT results are often evaluated by selecting the unprocessed thermal image with the maximum contrast between the sound and defective area. Recently, many signal processing algorithms have been applied to raw thermal images to access the defects.

### 3.1. Thermography Signal Reconstruction

Thermography signal reconstruction (TSR) is a renowned signal processing technique to improve and distinguish pulsed thermographic images, sorting out spatial and temporal non-uniform noise and suppressing the later one. Furthermore, it provides the log-log thermal response of each pixel of sequence with a polynomial of the nth degree. The logarithmic transform of Equation (2) can be expressed by Equation (3) [54,66,67]

(3) $$\ln\left(T\right)=\ln\left(\frac{Q}{e}\right)-\frac{1}{2}\ln\left(\pi t\right)\;={\;a}_{0}+a_{1}\ln\left(t\right)+a_{2}{\left[\ln\left(t\right)\right]}^{2}+\ldots +a_{n}{\left[\ln\left(t\right)\right]}^{n}$$

where T is the increment in temperature as a function of time and $a_{0}$, $a_{1}$,…,$a_{n}$ are the polynomial coefficients. In NDT, fifth or sixth order polynomial was found effective in temporal noise reduction and the optimum degree of the polynomial in use was found up to ninth order. The first log-time derivative dln(T)/dln(t) and the second derivative $d^{2}\ln\left(T\right)/{\mathrm{dln}}^{2}\left(t\right)$ of the polynomial enhanced the defect detectability and SNR [68,69].

### 3.2. Pulsed Phase Thermography

In pulse phase thermography, the discrete Fourier transform is applied to transform each pixel of thermal image sequence from a time domain to frequency domain and can be expressed by Equation (4) [52,53,70]:

(4) Fn=∆t∑k=0N−1T(k∆t)expj2πnkN=Ren+Imn

where R_e_ and I_m_ are, respectively, the real and imaginary parts of the transform sequence, F_n_, N is the number of thermal images, j^2^ = −1 is the imaginary number, and Δt is the sampling time interval. The phase component (∅) of the transformed data can be obtained using Equation (5) [53,70].

(5) ∅n=tan−1(ImnRen)

### 3.3. Principle Component Thermography

Principle component thermography (PCT) facilitates in the reduction of undesirable data while preserving the major features of thermal image sequences. The recorded 3-D matrix of the thermal image sequence is transformed into a 2-D matrix, A of dimension MxN. Then, the singular value decomposition method is used to extract the spatial and temporal information and can be expressed by Equation (6) [55,71].

(6) $$A={\mathrm{USV}}^{T}$$

where U is the orthogonal matrix of dimension MxN and comprises a set of empirical orthogonal functions (EOFs) representing spatial variation. S is a diagonal matrix of dimension NxN and possesses the singular values of matrix A on the diagonals. V^T^ is the transpose matrix of the NxN orthogonal matrix representing characteristic time.

## 4. Methods and Materials

### 4.1. Test Sample

**Figure 3:**
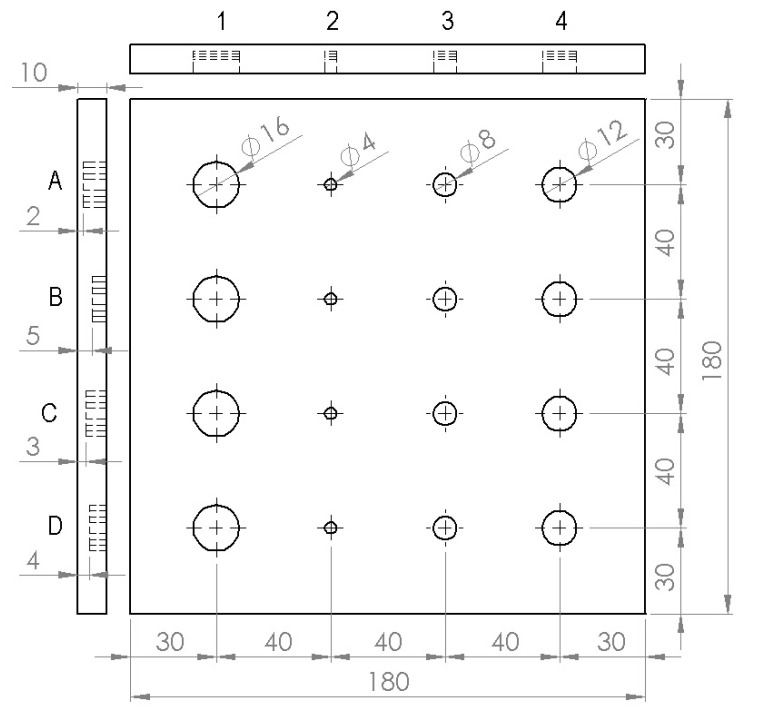
Schematic illustration of austenitic stainless steel (SUS 316) sample along with the geometry and location of artificial flat-bottomed holes representing locally wall thinned areas of different sizes at varying depth levels.

**Figure 4:**
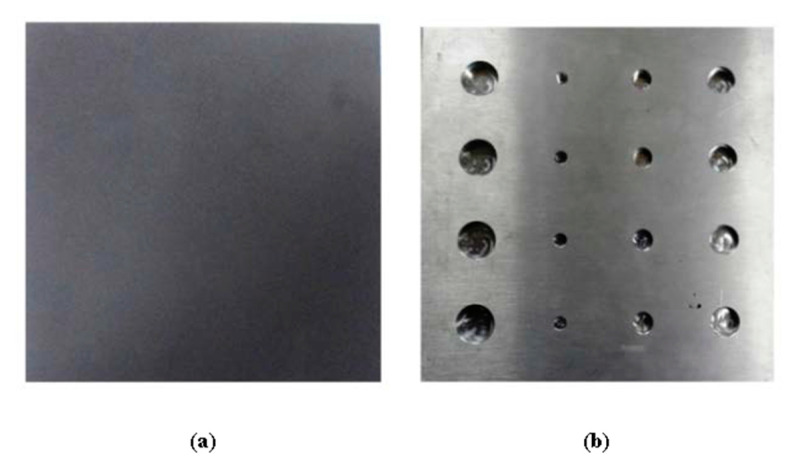
Photographs of the sample used for the study, (**a**) front side with black paint and (**b**) rear side with flat-bottomed holes.

A sample made of austenitic stainless steel (SUS 316) with the flat-bottomed holes (FBH) was considered to simulate wall thinning defect. Figure 3 depicts the schematic illustration of the sample, along with the geometry and location of artificial FBH. It is a square-shaped plate with dimensions 180 × 180 mm and thickness of 10 mm. Artificial FBHs of different sizes at varying depth levels were milled at the rear surface to evaluate the detection capability of the proposed technique. The FBHs in each column represents the defects with a constant diameter, but of different depth from uppermost to lowermost row. Referring to Figure 3, ‘A’, ‘B’, ‘C’, and ‘D’ represent defects along the horizontal rows and have different defect diameters. Similarly, ‘1’, ‘2’, ‘3’, and ‘4’ represent the defect size along with vertical columns and have different depths. All the sixteen defects are indexed with a distinctive ‘Defect ID’ using these representations. For instance, defect ID of defect in the first row and the first column is ‘A_1_’ which has a diameter of 16 mm and depth of 2 mm; defect ID of defect in second row and second column is ‘B_2_’ which has a diameter of 4 mm and depth 5 mm; defect ID of defect in third row and third column is given as ‘C_3_’ which has a diameter of 8 mm and depth of 3 mm; defect ID of defect in the last row and the last column is given as ‘D_4_’ which has a diameter of 12 mm and depth of 4 mm, and so on. The front side of the sample was painted black using KRYLON flat paint having an emissivity of 0.95 to create a uniform emissive surface. Figure 4 depicts the photographs of the sample considered for the study.

### 4.2. Experimentation

In this research, the experimental setup consisted of a test sample, short and high power heating source with the system controller, an infrared camera and a computer. As a thermal excitation source, a flash lamp (Universal BALCAR, Rungis, Paris, France) of power 6400 W-s was used and controlled by a power box (BALCAR Light System, Nexus A 6400, France). An infrared camera (SC655, FLIR Systems, Danderyd, Sweden) with a spectral range 7.5–13 μm, thermal sensitivity (NETD) <50 mk, accuracy ±2 °C, and a 640 × 480 focal plane array detector with the frame rate of 50 Hz was used to record the thermal response of the test sample. A computer (MSI GE620DX) was used to control the infrared camera and to store the experimental data. FLIR R&D software was used for the pre-processing of raw thermal data. FLIR R&D also includes the features of camera control, high-speed data recording, image analysis, and data sharing. Data recording options include start time, end time, and the number of frames to acquire.

## 5. Results and Discussions

The experiment was conducted in a dark and closed chamber at Thermal Design and Infrared Laboratory of Kongju National University, Korea. The temperature of the chamber was set 22 °C and relative humidity was maintained 48%. To evaluate the thermal response of the front surface, the test sample was set upright, and reflection mode was chosen. The sample front surface was excited by a pulse heat for a duration of approximately 10 milliseconds. Then, the thermal response of the sample was recorded for 5 s.

**Figure 5:**
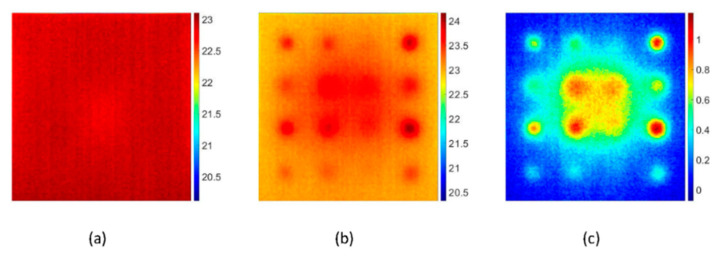
Experimental pulsed thermal images on the front surface of the flat-bottomed holes sample after pre-processing, (**a**) thermal image at time 0 s before the application of pulse heating, (**b**) thermal image at time 0.1 s after the deposition of heat flux where defect exhibit the higher signal-to-background contrast and (**c**) thermal image which is result of subtracting Figure 5a from Figure 5b [Frequency = 50 Hz, number of frames = 250, truncation window = 5 s]. Temperature scales are in degree Celsius.

**Figure 6:**
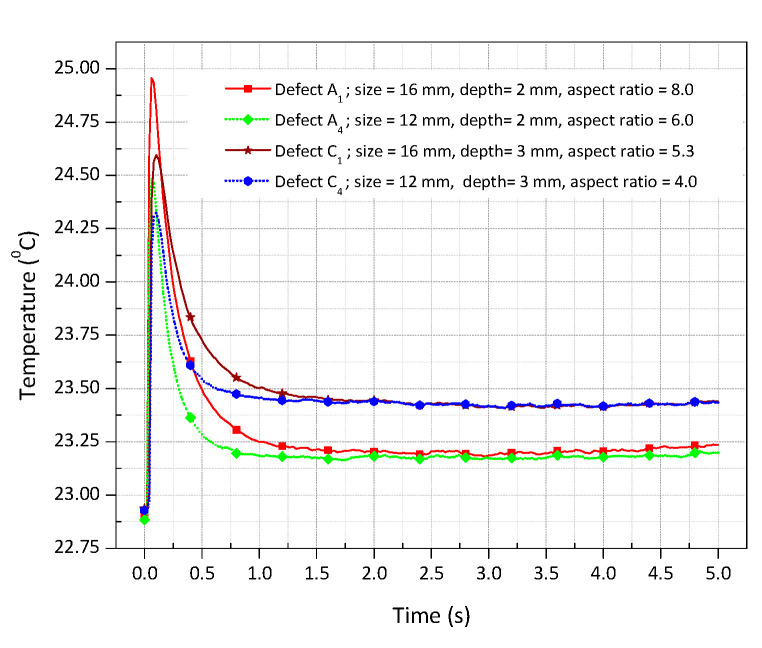
Defect detection from temperature profiles of flat-bottomed holes in a steel plate subjected to pulse heating [Frequency = 50 Hz, number of frames = 250, truncation window = 5 s].

Figure 5a depicts the thermal image at time 0 s recorded before the application of a pulse heating. Figure 5b depicts the thermal image at time 0.1 s recorded after the deposition of heat flux where the detected defects exhibit the higher signal-to-background contrast without performing any data processing techniques. Figure 5c depicts the thermal image which is a result of subtracting Figure 5a from Figure 5b. The results showed that the shallower defects with larger size were detected clearly, whereas the deeper defects with smaller size were detected faintly. To investigate the effects of defect geometry on the applied heat flux, the thermal response of the defects with different aspect ratio (ratio of diameter to depth) was analyzed. Defects A_1,_ A_4_, C_1,_ and C_4_ with an aspect ratio of 8.0, 6.0, 5.3, and 4.0, respectively, were chosen for the investigation. The ROI of 4 × 4 pixels at the centre of each defect was considered, and the average mean temperature was measured. Figure 6 depicts the thermal response of the selected defects concerning the time of 5 s. It was observed that the temperature attained by the sample was faster than its decay because of short impulse time. As time passed, the temperature on the surface tends to reach equilibrium. It was worth noting that the shallower defect A_1_ with a high aspect ratio causes significant temperature difference when compared with other defects with a smaller aspect ratio. The variation in the surface temperature distribution was enough to distinguish the defects. However, the processing of thermal images was done to reduce noise and enhance the defect detectability. The algorithms stated in Section 3 were compiled by using MATLAB^®^ R2019a.

### 5.1. Thermal Signal Reconstruction Results

**Figure 7:**
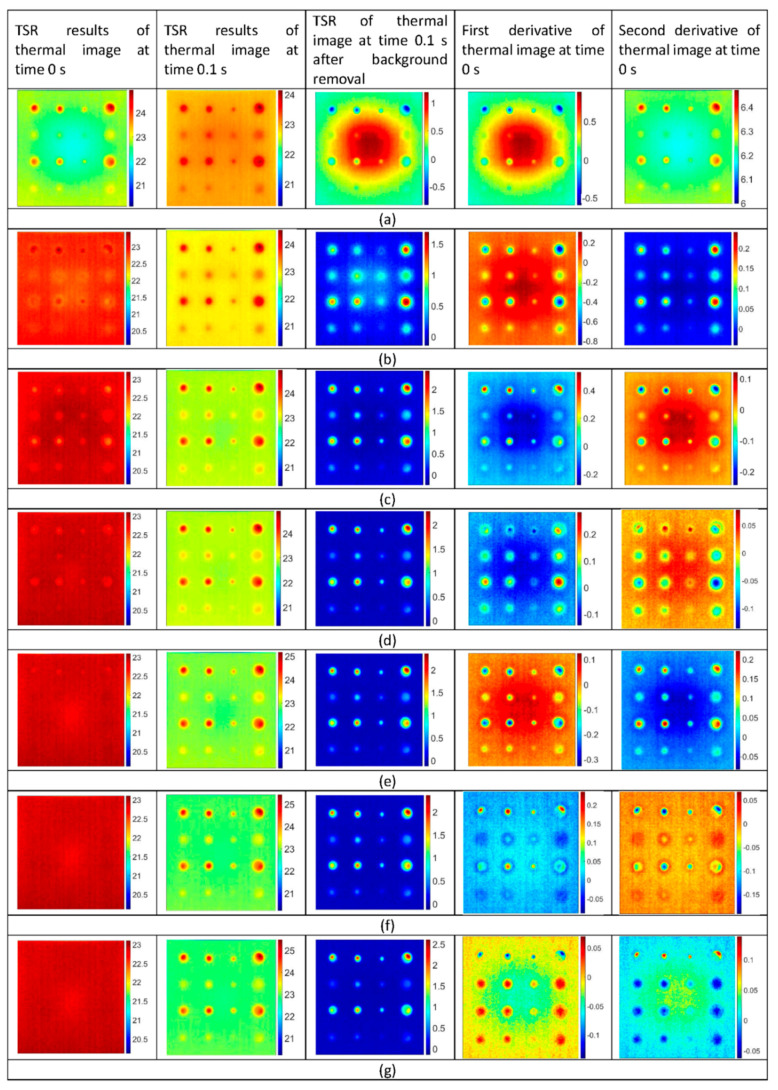
Comparison of experimental pulsed thermography images processed with thermal signal reconstruction for the times corresponding to order of polynomial fitting, (**a**) 2nd order, (**b**) 3rd order, (**c**) 4th order, (**d**) 5th order, (**e**) 6th order, (**f**) 7th order and (**g**) 8th order [Frequency = 50 Hz, number of frames = 250, truncation window = 5 s]. Temperature scales are in degree Celsius.

**Figure 8:**
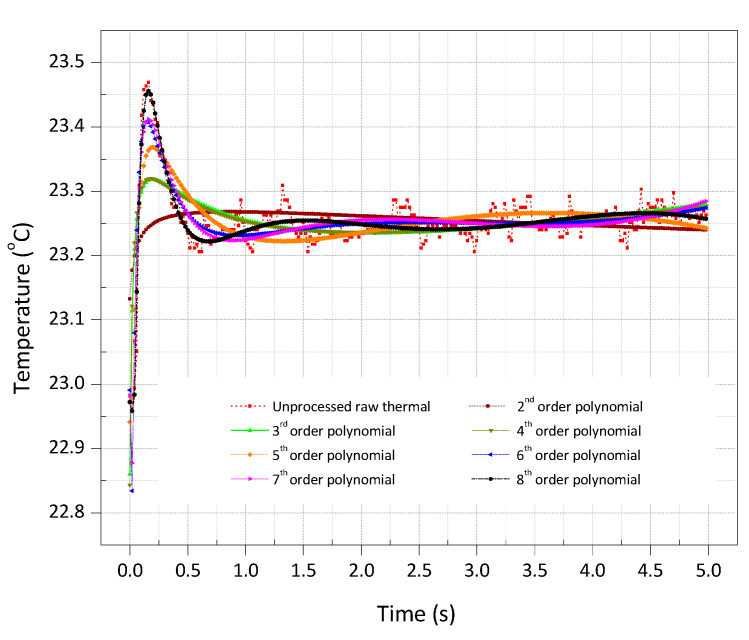
Residuals along the time as a function of the numbers of coefficient in the regression of a flat-bottomed hole of size 16 mm and depth 2 mm [Frequency = 50 Hz, number of frames = 250, truncation window = 5 s].

The TSR processing was applied to each pixel of the raw thermal images to reconstruct the logarithmic time dependence and the corresponding first and second derivatives. The logarithmic polynomial coefficients up to 8th degree were evaluated. To perceive the effects of TSR process, the thermal image at time 0 s (Figure 5a) and the thermal image at time 0.1 s (Figure 5b) were selected for the further investigation. Figure 7 depicts the results of TSR processing of the selected thermal images. In Figure 7, the first column depicts the TSR results for the thermal image at time 0 s; the second column depicts the TSR results for the thermal image at time 0.1 s; the third column depicts the thermal image acquired after subtracting the thermal image of first column from the second column; the fourth column depicts the first derivative of the first column thermal image and 5th column depicts the second derivative of the second column thermal image; for different degrees of polynomial coefficients. It was observed that the selection of an appropriate polynomial degree in the regression process plays a vital role to fit the thermal data. To measure the quality of TSR fitting, root mean square error (RMSE) for each degree of polynomial fitting was evaluated. For this purpose, the defect A_1_ with a high aspect ratio of 8 was considered, and the temperature at the centre was measured. Figure 8 depicts the residuals, and Table 1 presents the RMSE as a function of the numbers of coefficients for the defect A_1_. It was found that the TSR regression with two coefficients has large residuals at the beginning of the cooling process. With three coefficients, the residuals and the RMSE began to reduce and improved with the increasing one. On the contrary, the first and second derivatives provided the best results with just three degrees of a polynomial function, as noticed in Figure 7b.

### 5.2. Pulsed Phase Thermography Results

**Figure 9:**
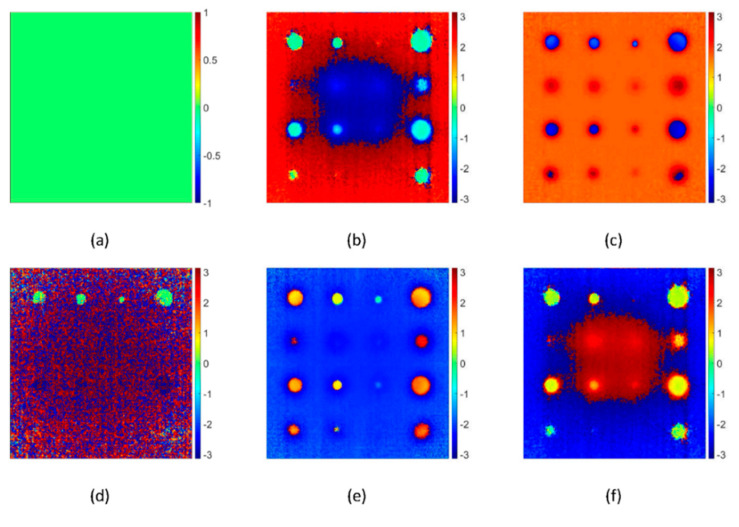
Phase images obtained with pulsed phase thermography for a steel plate with flat bottomed holes after pulse heating at different frequency spectra, (**a**) 0.2 Hz, (**b**) 0.4 Hz, (**c**) 8 Hz, (**d**) 25 Hz (**e**) 42.6 Hz and (**f**) 50 Hz, number of frames =250 frames, truncation window 5 s. Phase angle scales are in radian.

Figure 9 depicts the results of PPT concerning frequency spectra. It was observed that defect detectability with PPT is dependent on the chosen frequency spectra. Figure 9a depicts the phase image at frequency 0.2 Hz, where no defects were detected. Figure 9b depicts the phase image at frequency 0.4 Hz, where defects started to be detected but affected by the noise. Figure 9c depicts the phase image at frequency 8 Hz, where a maximum number of defects were detected with high phase contrast. Figure 9d represents the phase image at a frequency of 25 Hz, where most of the defect information was lost. Figure 9e depicts the phase image at frequency 42.6 Hz, where a maximum number of defects were detected with high phase contrast. It was worth noting that phase inversion takes place at frequency 42.6 Hz where the defects which were blue at frequency 8 Hz changed to orange. Figure 9f depicts the phase image at frequency 50 Hz, where defects were affected by the noise again.

**Figure 10:**
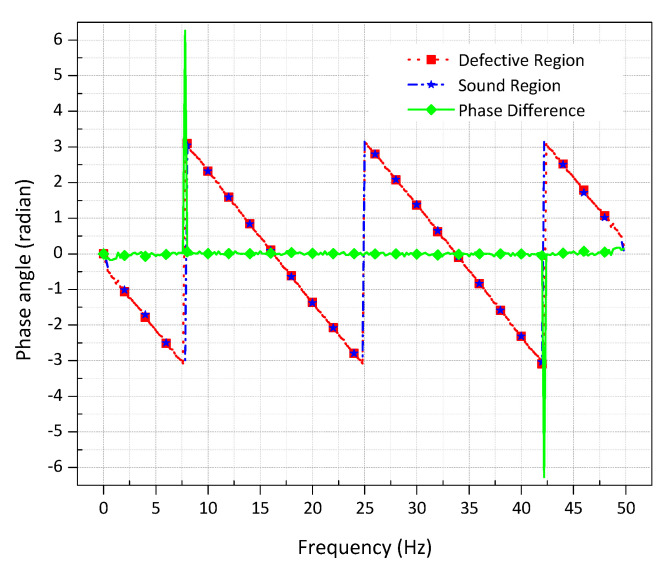
Phase profiles of defective and sound regions for a flat-bottomed hole of size 16 mm and depth 2 mm on a steel plate obtained by pulse phase thermography concerning frequency; frequency rate =50 Hz, number of frames =250 frames, truncation window 5 s.

To select the most appropriate frequency for high defect detectability and noise reduction, phase contrast analysis for the defect A_1_ was performed. For this purpose, two ROIs of 4 × 4 pixels, one at the centre of the defect and another in the adjacent sound region were considered. Figure 10 depicts the phase contrast for the defect A_1_ concerning frequency spectra. It was found that there exist two favourable frequencies where the defect exhibits maximum phase contrast. Defect exhibits the maximum positive phase contrast at a frequency of 8 Hz and maximum negative phase contrast at a frequency of 42.6 Hz.

### 5.3. Principle Component Thermography Results

**Figure 11:**
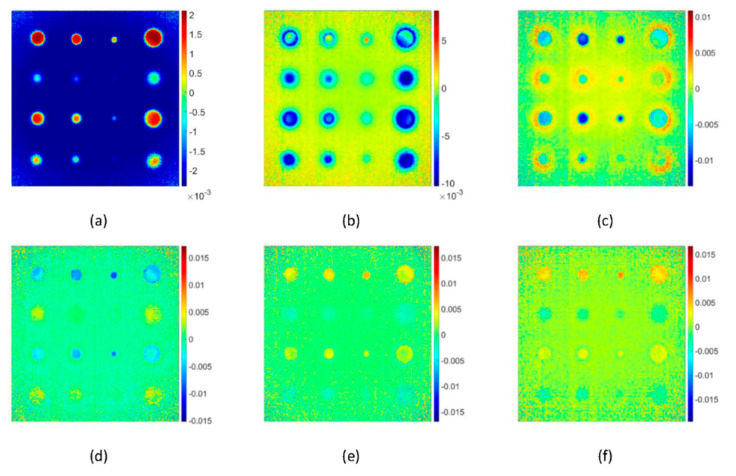
Experimental pulsed thermography images processed by principle component thermography concerning EOFs, (**a**) 1st EOF, (**b**) 2nd EOF, (**c**) 3rd EOF, (**d**) 4th EOF, (**e**) 5th EOF and (**f**) 6th EOF. The scales are in “digital level” units.

Figure 11 shows the results of PCT concerning EOFs. Unlike in TSR and PPT, the results of the PCT are less dependent on the number of frames and frequency spectra. The most meaningful information was found in the first two EOFs.

### 5.4. Comparison of Processing Techniques

The performance of all the three processing techniques was measured in terms of enhanced defect detectability and SNR. The optimum results provided by each technique were considered in the measurement. The results of TSR with the 8th degree of the polynomial fitting, the result of PPT at a frequency of 0.8 Hz and the result of PCT with 1st EOF were compared with the raw thermal image at time 0.1 s.

#### 5.4.1. Comparison Based on Defect Detectability

The raw thermal image at time 0.1 s (Figure 5b) can detect 8 out of 16 defects. The detected defects were of size 16 mm and 12 mm with an aspect ratio of more than 3. TSR with the 8th degree of polynomial fitting (Figure 7e) can detect 16 out of 16 defects. Even the smaller defects with a size 4 mm and an aspect ratio of 0.8 were also detected faintly. PPT at a frequency of 0.8 Hz (Figure 9c) and PCT with 1st EOF (Figure 11a) were able to detect 14 out of 16 defects. Both PPT and PCT techniques were found insensitive to the smaller defects of size 4 mm within an aspect ratio of 0.8–1.0.

#### 5.4.2. Comparison Based on Signal to Noise Ratio

Two ROIs of 4 × 4 pixels, one at the centre of the defect and another in the adjacent sound area, were considered for each defect. ROI in the defective area was considered as “signal” (DROI), and ROI in the sound area was considered as “noise” (SROI). Then, SNR was acquired by Equation (7) [72,73].

(7) $$\mathrm{SNR}=20\log_{10}\left(\frac{\left|{\mathrm{DROI}}_{\mathrm{mean}}-{\mathrm{SROI}}_{\mathrm{mean}}\right|}{\sigma }\right)$$

where DROI_mean_, SROI_mean_, and σ represent, respectively, the arithmetic mean of ROI in the defective area, the arithmetic mean of ROI in the sound area, and standard deviation of ROI in the sound area.

The results obtained using Equation (7) to the data are presented in Table 2. The results demonstrated that all three signal processing techniques greatly improved the SNR. PPT and PCT dominated the TSR in terms of SNR. It was worth noting that PCT dominates PPT for the shallower defects of depth 2 and 3 mm, whereas PPT dominates for the deeper defects of depth 4 mm and 5 mm. For example, in Table 2 for shallower defect A_1_; the raw thermal image provided the SNR of 33.26 dB; TSR provided the SNR of 40.46 dB, which is an increment of 21.64%; PPT provided the SNR of 45.24 dB, which is an increment of 33.26%; PCT provided the SNR of 47.78 dB which is an increment by 43.65%; when compared with the raw thermal image. Similarly, for the deeper defect B_1_; the raw thermal image provided the SNR of 19.18 dB; TSR provided the SNR of 31.18 dB, which is an increment of 62.56%; PPT provided the SNR of 46.28 dB, which is an increment of 141.29%; PCT provided the SNR of 41.6 dB which is an increment of 116.89%; when compared with the raw thermal image.

## 6. Conclusions and Future Works

In this work, pulsed thermography was applied to a steel structure with the aim to detect wall thinning defects. The fundamental concepts of TSR, PPT, and PCT have been reviewed for the processing and analysis of thermal images. The performance of each signal processing technique was evaluated in terms of enhanced defect detectability and SNR. Based on the results, it was determined that a considerable improvement in the defect detection capability and SNR values could be obtained after the implementation of techniques.

TSR provided the less noisy image when compared with the raw thermal image and bring essential improvements in defect detectability due to an increase of temporal and spatial resolution and the ability to produce time derivative images. However, variation in the results in different instants of time or frames could be disadvantages of TSR. PPT has been found highly sensitive to the defect depth and provided the best SNR for the smaller and deeper defects. However, the results of PPT are also sometimes difficult to handle since it also provides several data in different frequency spectra. The PCT has been found useful for detecting the larger and shallower defects. The independence of the results on the number of frames was found as the major advantage of the PCT.

Future work will be directed towards implementing pulsed thermography signal processing techniques to detect real defects in the pipe structure. Furthermore, the maximum depth detection capability of pulsed thermography will be investigated. Additionally, TSR based analysis will be done in high order polynomial fitting. Finally, the error analysis techniques will also be considered for the obtained results.

## Figures and Tables

**Table 1:** Root mean square error as a function of the numbers of coefficient in the regression for a flat-bottomed hole of size 16 mm and depth 2 mm.

Degree of Polynomial Coefficient	Root Mean Square Error
2nd	0.0503
3rd	0.0396
4th	0.0395
5th	0.0326
6th	0.0263
7th	0.0255
8th	0.0200

**Table 2:** SNR values for each processing techniques and flat-bottomed holes.

Defect ID.	SNR			
	Raw Image	TSR	PPT	PCA
A_1_	33.26	40.46	45.24	47.78
A_2_	-	32.39	42.15	44.56
A_3_	-	39.18	43.92	47.15
A_4_	28.17	39.47	44.56	47.72
B_1_	19.18	31.18	46.28	41.60
B_2_	-	26.15	-	-
B_3_	-	31.75	33.65	31.90
B_4_	12.19	28.78	40.76	38.85
C_1_	32.14	38.98	46.55	46.58
C_2_	-	32.29	36.52	34.91
C_3_	-	39.47	45.41	45.58
C_4_	28.39	38.28	45.90	46.76
D_1_	23.96	28.78	47.12	44.52
D_2_	-	15.62	-	-
D_3_	-	27.82	45.91	40.26
D_4_	21.44	27.64	47.10	44.16

Note: The symbol ‘-’ is the representation of non-detected defects.
